# Association between exposure to urinary metal and all-cause and cardiovascular mortality in US adults

**DOI:** 10.1371/journal.pone.0316045

**Published:** 2024-12-27

**Authors:** Ting Cheng, Dongdong Yu, Geng Li, Xiankun Chen, Li Zhou, Zehuai Wen

**Affiliations:** 1 Second Clinical College of Guangzhou University of Chinese Medicine, Guangzhou, China; 2 First Affiliated Hospital of Anhui University of Chinese Medicine, Hefei, China; 3 Guangdong Provincial Hospital of Chinese Medicine (Second Affiliated Hospital of Guangzhou University of Chinese Medicine), Guangdong Provincial Academy of Chinese Medical Sciences, Guangzhou, China; 4 Science and Technology Innovation Center of Guangzhou University of Chinese Medicine, Guangzhou, China; Instituto Nacional de Cardiologia Ignacio Chavez, MEXICO

## Abstract

**Background:**

Further evidence is required regarding the influence of metal mixture exposure on mortality. Therefore, we employed diverse statistical models to evaluate the associations between eight urinary metals and the risks of all-cause and cardiovascular mortality.

**Methods:**

We measured the levels of 8 metals in the urine of adults who participated in the National Health and Nutrition Examination Survey (NHANES) from 1999 to 2018. Based on follow-up data, we determined whether they died and the reasons for their deaths. We estimated the association between urine metal exposure and all-cause mortality using Cox regression, weighted quantile sum (WQS) regression, and Bayesian kernel machine regression (BKMR) models. Additionally, we used a competing risk model to estimate the relationship between metal exposure and cardiovascular mortality.

**Results:**

Among the 14,305 individuals included in our final analysis, there were 2,066 deaths, with 1,429 being cardiovascular-related. Cox regression analysis showed that cobalt (Co) (HR: 1.21; 95% CI: 1.13, 1.30) and antimony (Sb) (HR: 1.26; 95% CI: 1.12, 1.40) were positively associated with all-cause mortality (all *P* for trend <0.001). In the competing risk model, Co (HR: 1.29; 95% CI: 1.12, 1.48), lead (Pb) (HR: 1.18; 95% CI: 1.03, 1.37), and Sb (HR: 1.44; 95% CI: 1.18, 1.75) were significantly associated with an increased risk of cardiovascular mortality (all *P* for trend <0.001). Sb, Pb, cadmium (Cd), and molybdenum (Mo) had the highest weight rankings in the final WQS model. All metals showed a complex non-linear relationship with all-cause mortality, with high posterior inclusion probabilities (PIPs) in the final BKMR models.

**Conclusions:**

Combining all models, it is possible that Sb may have a more stable impact on all-cause and cardiovascular mortality. Meaningful metal effects in individual statistical models still require careful attention.

## Introduction

Metal pollution is considered one of the most common byproducts of industrialization and urbanization. The general public can be exposed to various metals through mediums such as water, air, soil, and food [1]. An increasing amount of epidemiological evidence suggests that exposure to metals increases the risk of various diseases, such as cardiovascular disease, arteriosclerosis, infertility, and kidney stones [2–5]. In addition, some studies have found that the levels of metals in blood and urine in the human body can partly predict the risk of all-cause, cardiovascular, and cancer mortality [6–9]. Most of the early literature is limited to analyzing the effects of individual chemicals on human health or death [10,11]. The effects of different metal exposures on the human body may be cumulative, synergistic, antagonistic, or indirectly complex, requiring more evidence to support each other [12]. Even though a few studies have begun to focus on the effects of metal mixtures on various diseases [12–15], however, extending it to survival analysis is rare. Additionally, due to competitive risk bias, traditional survival analysis methods used in the early stages may overestimate the impact of metals on specific mortality risk [16,17]. The different exposure patterns, high correlations, and complex interactions of metals in the environment require more statistical strategies to evaluate their combined effects [18]. Currently, many new statistical methods have been successfully introduced into the field of environmental health [19,20], previous studies exploring mixture exposure have shown that combining multiple methods can lead to more comprehensive and reliable conclusions [7]. It is difficult to establish a set of standard criteria for determining which statistical method is most suitable for a specific study. Therefore, there is still insufficient evidence on the combined effects of different metals on mortality.

This study employed data from the National Health and Nutrition Examination Survey (NHANES) carried out from 1999 to 2018 to evaluate the connection between the presence of eight heavy metals in the urine and the all-cause mortality rate for the general population in the United States (US). We employed three different models, specifically Cox regression, weighted quantile sum (WQS) regression, and Bayesian kernel machine regression (BKMR), to assess this association. Moreover, we examined the link between metal exposure and the risk of cardiovascular mortality using a competing risk model. Subsequently, we interpreted the results from multiple models jointly to further explore the relationship between mixed metal exposure and mortality risk. These findings may offer novel suggestions for longitudinal epidemiological and experimental investigations.

## Materials and methods

### Study population

**Fig 1:**
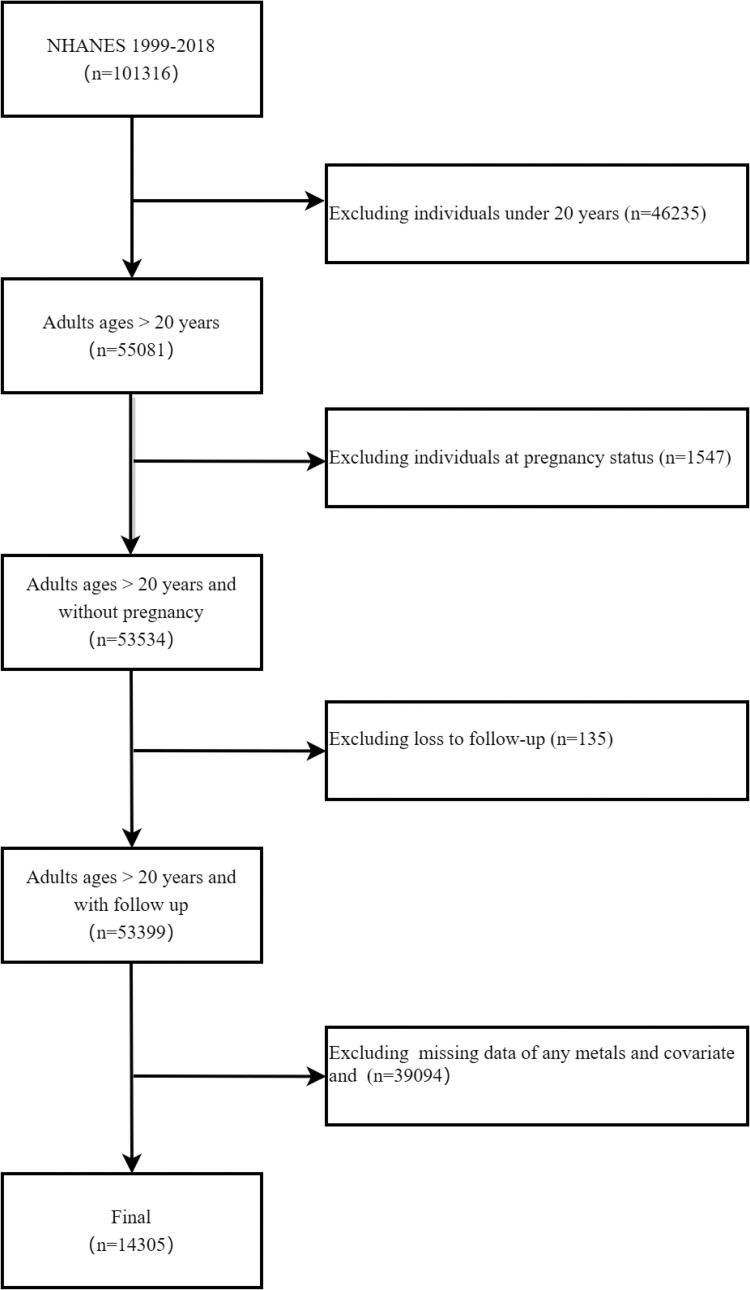
Selection criteria for participants in this study.

NHANES is an ongoing national cross-sectional survey, and its data can be found on the website of the Centers for Disease Control and Prevention (CDC) in the US (https://wwwn.cdc.gov/nchs/nhanes/Default.aspx) [21]. The research protocol has been approved by the Research Ethics Review Committee of the National Center for Health Statistics (NCHS). All participants provided written consent at the time of recruitment. In this study, we included 101316 data from 10 survey cycles conducted between 1999 and 2018. Exclusion criteria include: (a) age < 20 years; (b) pregnancy status; (c) loss to follow-up; (d) missing data of any covariate. A total of 14,305 participants were ultimately included in the final analysis (Fig 1). As this study is a retrospective analysis and poses no risk of exposure to personally identifiable information, no additional ethical review or informed consent is required.

### Measurement of urinary metal and covariation assessment

The data for 8 urine metals, namely cadmium (Cd), cobalt (Co), lead (Pb), barium (Ba), antimony (Sb), cesium (Cs), molybdenum (Mo), and thallium (Tl), were obtained from NHANES 1999–2018. These metals in urine samples were primarily measured by using the Inductively Coupled Plasma Mass Spectrometry (ICP-MS). The final metal concentrations used in the analysis were obtained by dividing the metal concentration (μg/L) by the urine creatinine concentration (mg/dL) [22].

We have taken into account the covariates that are most likely to affect metal exposure and mortality rates, including gender, age, race, body mass index (BMI), marital status, education level, family poverty income ratio (PIR), smoking, alcohol consumption, health insurance [12,23]. PIR refers to the ratio of household income to the federal poverty line, used to measure the relationship between household income and poverty level [24]. Smoking is defined as having smoked at least 100 cigarettes in one’s lifetime. According to the categorization of alcohol consumption levels by the United States Department of Agriculture, drinking is divided into five categories: never, former, light, moderate, and heavy [25]. We also include self-reported cardiovascular diseases (if including any of congestive heart failure, coronary heart disease, angina or myocardial infarction), stroke (yes or no), cancer (if including any type of cancer), hypertension (yes or no) and diabetes (yes or no).

### Outcomes

The public use mortality files from NHANES offer mortality data recorded in the survey registration up to December 31, 2019. These files enable tracking of participant deaths and identification of specific causes. Starting from 1998, the death codes adhere to the guidelines of the 10th edition of the International Classification of Diseases, Injuries, and Causes of Death (ICD-10) [26]. Determine alive or deceased based on the variable MORTSTAT (final mortality status). The mortality specifically related to cardiovascular diseases (coded I00-I09, I11, I13 and I20-I51) was identified based on the ICD-10. For participants who did not experience the event during the study period, their follow-up time was considered right-censored. Similarly, all missing follow-up data were treated as right-censored.

### Statistical analysis

All analyses were conducted using R 4.2 (the R Foundation for Statistical Computing, Vienna, Austria). Chi-square tests or t-tests were used to assess participants’ baseline characteristics. Categorical variables were represented as numbers (n) and percentages (%), while continuous variables were represented as means and the 25th and 75th percentiles (P25, P75). This study used creatinine-corrected metal concentrations to analyze the relationship between heavy metal exposure levels and mortality risk, in order to reduce the impact of urine dilution on the measurements [12]. Due to the severely skewed concentrations of these eight chemicals, the standardized data were ln-transformed when treated as continuous variables to approximate a normal distribution. Firstly, we analyzed the Pearson correlation coefficients between the ln-transformed concentrations of the eight metals and the trends of individual metal concentrations in each survey period.

Only Co, Mo, Pb, and Sb passed the proportional hazards (PH) assumption (S1 Table). Cox regression analysis was used to examine the relationship between individual metals and all-cause mortality, and individual metals were categorized into four quartiles (i.e., were split by P25, P50 and P75 into Q1, Q2, Q3, and Q4, respectively) to calculate Hazard Ratio (HR) and *P* for trend.

Competing risks refer to events that may hinder the occurrence of the primary event of interest, as these events can prevent the occurrence of the event of interest or alter the probability of the primary endpoint occurring [16]. For example, when studying cardiovascular mortality, deaths due to non-cardiovascular causes are considered competing risks. When estimating the incidence of the target outcome, analysts should use the cumulative incidence function [27]. Considering that cardiovascular mortality may be masked by other causes, a competing risk model was used to assess the impact of individual metals on the risk of cardiovascular death.

The weighted quantile sum (WQS) regression model constructs a weighted index ranging from 0 to 1, reflecting the contribution of each metal in the mixture [28]. It is used to estimate the combined impact of all environmental chemical exposures on the outcome and can be employed to test the association between the index and the dependent variable or outcome [29]. Consequently, the relative strength of weights assigned to each variable in the model allows for the assessment of each environmental chemical’s contribution to the overall index effect, thus identifying important chemicals within the mixture [12]. It is important to note that the WQS regression limits the effect of a single metal on mortality risk to one direction. In this study, we used the R package “gWQS” with family = binomial to construct the WQS index for heavy metal exposure, analyzing the combined impact of metal mixture components on all-cause risk, and used 40% of the data as a training set and the remaining data as a test set [30]. We performed 1000 bootstrap samples in the multivariable regression model and applied regularization constraints in the optimization function for weight estimation.

To evaluate the combined impact of metal mixtures on all-cause mortality, we used the “Bayesian kernel machine regression (BKMR)” package and adopted default priors for 10,000 iterations [31]. We analyzed the following three exposure-response functions: 1) Univariate exposure-response function: Assessed the impact of individual metal exposure on all-cause mortality risk. 2) Cumulative effect at different percentiles: Compared the cumulative effect of metal mixtures at different percentiles on cardiovascular disease (CVD) risk. 3) Bivariate exposure-response function: Evaluated the impact of one metal on all-cause mortality risk while fixing the other metals at different percentiles [12,28]. Additionally, we calculated the posterior inclusion probability (PIP) for each metal to identify those with the most significant impact on all-cause mortality risk.

For all statistical methods mentioned above, we progressively built four models to assess the potential confounding effects of different covariate combinations. Model 0 was a crude model, Model 1 adjusted for age, sex, and race. Model 2 additionally considered marital status, PIR, education, BMI, smoking, drinking, and health insurance. In Model 3, we further adjusted for the combined presence of diabetes, stroke, cancer, CVD, and hypertension. A two-sided *P*-value less than 0.025 was considered statistically significant.

## Results

### Population characteristics

During the 10 survey cycles, a total of 14,305 participants were included, with 2,066 deaths recorded as all causes (Table 1). Among these deaths, 1,429 were due to cardiovascular reasons. The median follow-up time was 101 months, with the longest follow-up period being 249 months. The age range of the participants was between 20 and 85 years, with males accounting for 51.61% of the total. The majority of deaths due to all causes were in individuals over 60 years old, male, white ethnicity and of highly educated. The cumulative incidence rates of all-cause and cardiovascular deaths can be seen in S1 Fig.

**Table 1:** Basic characteristics of subjects (NHANES1999-2018).

Characteristic	Overall, N = 14,305	Assumed alive, N = 12,239	Assumed deceased, N = 2,066	*P*
**Year**				<0.001
1999–2000	768(5.37%)	523 (4.27%)	245 (11.86%)	
2001–2002	1231 (8.61%)	893 (7.30%)	338 (16.36%)	
2003–2004	1177 (8.23%)	843 (6.89%)	334 (16.17%)	
2005–2006	1249 (8.73%)	993 (8.11%)	256 (12.39%)	
2007–2008	1528 (10.68%)	1,266 (10.34%)	262 (12.68%)	
2009–2010	1624 (11.35%)	1,422 (11.62%)	202 (9.78%)	
2011–2012	1823 (12.74%)	1,632 (13.33%)	191 (9.24%)	
2013–2014	2008 (14.04%)	1,869 (15.27%)	139 (6.73%)	
2015–2016	1834 (12.82%)	1,754 (14.33%)	80 (3.87%)	
2017–2018	1063 (7.43%)	1,044 (8.53%)	19 (0.92%)	
**Age**				<0.001
20–39	4739 (33.13%)	4660 (38.08%)	79 (3.82%)	
40–59	4820 (33.69%)	4447 (36.33%)	373(18.05%)	
≥60	4746 (33.18%)	3132 (25.59%)	1614 (78.12%)	
Sex				<0.001
Female	6922 (48.39%)	6073 (49.62%)	849 (41.09%)	
Male	7383 (51.61%)	6166 (50.38%)	1217 (58.91%)	
**Race/Ethnicity**				<0.001
Non-Hispanic White	6644 (46.45%)	5387 (44.02%)	1257 (60.84%)	
Non-Hispanic Black	3057 (21.37%)	2598 (21.23%)	459 (22.22%)	
Mexican American	2226 (15.56%)	2021 (16.51%)	205 (9.92%)	
Other	2378 (16.62%)	2233 (18.24%)	145 (7.02%)	
**Marital status**				<0.001
Alone	5761 (40.27%)	4766 (38.94%)	995 (48.16%)	
Married or partner	8544 (59.73%)	7473 (61.06%)	1071 (51.84%)	
**Education**				<0.001
Less than 9th grade	1533 (10.72%)	1153 (9.42%)	380 (18.39%)	
High school graduate	5638 (39.41%)	4680 (38.24%)	958 (46.37%)	
College graduate or above	7134 (49.87%)	6406 (52.34%)	728 (35.24%)	
**PIR**	2.09 (1.10, 3.97)	2.15 (1.11, 4.12)	1.72 (1.08, 3.18)	<0.001
**Health insurance**				<0.001
No	3116 (21.78%)	2919 (23.85%)	197 (9.54%)	
Yes	11189 (78.22%)	9320 (76.15%)	1869 (90.46%)	
**BMI (kg/m** ^**2**^ **)**				<0.001
<25	4281 (29.93%)	3622 (29.59%)	659 (31.90%)	
25–29.9	4844 (33.86%)	4103 (33.52%)	741 (35.87%)	
≥30	5180 (36.21%)	4514 (36.88%)	666 (32.24%)	
**Alcohol**				<0.001
Never	1864 (13.03%)	1545 (12.62%)	319 (15.44%)	
Former	2507 (17.53%)	1789 (14.62%)	718 (34.75%)	
Mild	4639 (32.43%)	4022 (32.86%)	617 (29.86%)	
Moderate	2170 (15.17%)	1984 (16.21%)	186 (9.00%)	
Heavy	3125 (21.85%)	2899 (23.69%)	226 (10.94%)	
**Smoke**				<0.001
No	6887 (48.14%)	6127 (50.06%)	760 (36.79%)	
Yes	7418 (51.86%)	6112 (49.94%)	1306 (63.21%)	
**Follow-up time**	101.00 (60.00, 155.00)	105.00 (63.00, 160.00)	77.00 (42.00, 124.00)	<0.001
**Hypertension**				<0.001
No	8187 (57.23%)	7545 (61.65%)	642 (31.07%)	
Yes	6118 (42.77%)	4694 (38.35%)	1424 (68.93%)	
**Diabetes**				<0.001
No	12278 (85.83%)	10732 (87.69%)	1546 (74.83%)	
Yes	2027 (14.17%)	1507 (12.31%)	520 (25.17%)	
**Stroke**				<0.001
No	13772 (96.27%)	11921 (97.40%)	1851 (89.59%)	
Yes	533 (3.73%)	318 (2.60%)	215 (10.41%)	
**Cancer**				<0.001
No	12955 (90.56%)	11364 (92.85%)	1591 (77.01%)	
Yes	1350 (9.44%)	875 (7.15%)	475 (22.99%)	
**CVD**				<0.001
No	12,876 (90.01%)	11389 (93.05%)	1487 (71.97%)	
Yes	1429 (9.99%)	850 (6.95%)	579 (28.03%)	

HR: Hazard ratio; CI: Confidence intervals; BMI: Body mass index; PIR: Poverty income ratio; Cardiovascular diseases: Doctor diagnosed with any of the following—congestive heart failure, coronary heart disease, angina, or myocardial infarction; Stroke: Doctor diagnosed with a stroke; Cancer: Doctor diagnosed with any type of cancer; Diabetes: Doctor diagnosed with diabetes, glycohemoglobin HbA1c (%) > = 6.5, fasting glucose (mmol/L) > = 7.0, random blood glucose (mmol/L) > = 11.1, two-hour OGTT blood glucose (mmol/L) > = 11.1, or use of diabetes medication or insulin; Hypertension: Diagnosed with hypertension, currently taking antihypertensive medication, with an average systolic blood pressure > = 140 mmHg or average diastolic blood pressure > = 90 mmHg measured over three readings.

### Measurement of urinary metals and their correlation

The detection rates of metal content in urine are shown in S2 Table. Except for Sb, the detection rates of other metals are greater than 90%. The Pearson coefficients for the Ln transformation of metals show a moderate correlation between Cs and Tl (r = 0.58), while the correlations with other metals are relatively weak (S2 Fig). The concentration trends of individual metals during each survey period were reported in S3 Fig and S3 Table.

### Assess the association between urinary metal and all-cause and cardiovascular mortality

We used Cox regression analysis to examine the relationship between the levels of 4 urinary metals and the risk of all-cause mortality, as shown in Tables 2 and S4. In the crude model, Co, Mo, Pb and Sb were associated with an increased risk of all-cause mortality (all *P* for trend <0.001). After adjusting for all covariates in Model 3, Co (HR: 1.21; 95% CI: 1.13, 1.30) and Sb (HR: 1.26; 95% CI: 1.12, 1.40) remained positively associated with all-cause mortality (all *P* for trend <0.001).

**Table 2:** Cox regression analysis of the relationship between 4 metals and all-cause mortality.

	HR (95%CI)	Q1	Q2	Q3	Q4	*P* for trend
**Model 0**						
Co	1.36(1.27,1.46)	ref	1.03(0.91,1.16)	1.21(1.06,1.36)	1.52(1.35,1.71)	<0.001
Mo	1.16(1.09,1.24)	ref	0.98(0.86,1.11)	1.08(0.95,1.23)	1.26(1.12,1.43)	<0.001
Pb	1.76(1.65,1.87)	ref	1.43(1.19,1.71)	2.36(2.00,2.78)	3.14(2.68,3.69)	<0.001
Sb	1.35(1.21,1.51)	ref	1.16(1.00,1.35)	1.32(1.14,1.52)	1.49(1.30,1.71)	<0.001
**Model 1**						
Co	1.29(1.20,1.37)	ref	0.98(0.87,1.12)	1.22(1.07,1.38)	1.53(1.34,1.73)	<0.001
Mo	1.04(0.98,1.11)	ref	0.95(0.84,1.09)	1.12(0.98,1.27)	1.10(0.97,1.24)	0.028
Pb	1.20(1.12,1.30)	ref	0.84(0.70,1.01)	0.95(0.80,1.13)	1.07(0.91,1.27)	0.005
Sb	1.33(1.20,1.48)	ref	1.18(1.01,1.37)	1.39(1.21,1.61)	1.54(1.34,1.77)	<0.001
**Model 2**						
Co	1.26(1.17,1.35)	ref	0.95(0.84,1.08)	1.15(1.01,1.31)	1.41(1.24,1.60)	<0.001
Mo	1.05(0.99,1.12)	ref	0.94(0.82,1.07)	1.11(0.98,1.26)	1.08(0.95,1.22)	0.048
Pb	1.09(1.01,1.18)	ref	0.82(0.68,0.98)	0.89(0.75,1.06)	0.94(0.80,1.12)	0.385
Sb	1.28(1.15,1.43)	ref	1.07(0.92,1.25)	1.25(1.08,1.44)	1.39(1.21,1.59)	<0.001
**Model 3**						
Co	1.21(1.13,1.30)	ref	0.93(0.82,1.06)	1.10(0.96,1.25)	1.31(1.15,1.49)	<0.001
Mo	1.02(0.96,1.09)	ref	0.94(0.83,1.07)	1.07(0.94,1.22)	1.01(0.89,1.15)	0.425
Pb	1.12(1.03,1.20)	ref	0.88(0.73,1.05)	0.97(0.82,1.15)	1.03(0.87,1.22)	0.086
Sb	1.26(1.12,1.40)	ref	1.06(0.91,1.23)	1.25(1.08,1.44)	1.36(1.18,1.56)	<0.001

Co: Cobalt; Mo: Molybdenum; Pb: Lead; Sb: Antimony; HR: Hazard ratio; CI: Confidence Intervals; Q: Quartiles; BMI: Body mass index; PIR: Poverty income ratio; *P*-value less than 0.025; Q1, Q2, Q3 and Q4: Individual metals measurements were categorized by P25, P50 and P75 into Q1, Q2, Q3, and Q4, respectively.

Model 0: Adjusted for none.

Model 1: Adjusted for age, sex, race.

Model 2: Adjusted for age, sex, race, BMI, marital status, PIR, education, smoking, drinking, and health insurance.

Model 3: Adjusted for age, sex, race, BMI, marital status, PIR, education, smoking, drinking, health insurance, diabetes, stroke, cancer, CVD, and hypertension.

Table 3 presents the results of the competing risk model analyzing the relationship between the levels of 8 urinary metals and cardiovascular mortality. There were a total of 552 cardiovascular deaths. In the crude model, 5 metals (Cd, Co, Mo, Pb, Sb) were each positively associated with cardiovascular mortality (all *P* for trend <0.001), except for Ba, Tl, and Cs. After adjusting for all covariates in Model 3, Co (HR: 1.29; 95% CI: 1.12, 1.48), Pb (HR: 1.18; 95% CI: 1.03, 1.37), and Sb (HR: 1.44; 95% CI: 1.18, 1.75) remained significantly associated with an increased risk of cardiovascular mortality (all *P* for trend <0.001). These findings suggest that higher levels of Co, Pb, and Sb are robustly linked to an elevated risk of cardiovascular mortality, even when accounting for other competing risks and covariates.

**Table 3:** The relationship between 8 metals and cardiovascular mortality in the competing risk model.

		HR (95%CI)	*P*
**Model 0**	**n**		
Ba	552	0.85(0.76, 0.96)	0.006
Cd	552	1.88(1.63, 2.18)	<0.001
Co	552	1.46(1.28, 1.67)	<0.001
Cs	552	1.10(0.93, 1.31)	0.280
Mo	552	1.28(1.12, 1.46)	<0.001
Pb	552	1.69(1.53, 1.88)	<0.001
Sb	552	1.41(1.18, 1.69)	<0.001
Tl	552	0.77(0.57, 1.04)	0.088
**Model 1**			
Ba	552	0.87(0.78, 0.97)	0.0095
Cd	552	125(1.07, 1.45)	0.004
Co	552	1.36(1.19, 1.54)	<0.001
Cs	552	0.83(0.68, 1.01)	0.062
Mo	552	1.15(1.02, 1.30)	0.023
Pb	552	1.13(0.98, 1.31)	0.098
Sb	552	1.45(1.21, 1.74)	<0.001
Tl	552	0.88(0.67, 1.15)	0.34
**Model 2**			
Ba	552	0.90(0.81, 1.00)	0.05
Cd	552	1.20(1.01, 1.43)	0.037
Co	552	1.34(1.18, 1.53)	<0.001
Cs	552	0.95(0.78, 1.14)	0.580
Mo	552	1.15(1.02, 1.31)	0.025
Pb	552	1.12(0.96, 1.29)	0.140
Sb	552	1.47(1.21, 1.77)	<0.001
Tl	552	1.07(0.82, 1.38)	0.630
**Model 3**			
Ba	552	0.93(0.84, 1.03)	0.180
Cd	552	1.16(0.98, 1.38)	0.090
Co	552	1.29(1.12, 1.48)	<0.001
Cs	552	0.97(0.80, 1.17)	0.720
Mo	552	1.12(0.99, 1.27)	0.071
Pb	552	1.18(1.03, 1.37)	0.021
Sb	552	1.44(1.18, 1.75)	<0.001
Tl	552	1.06(0.82, 1.37)	0.630

Ba: Barium; Cd: Cadmium; Co: Cobalt; Cs: Cesium; Mo: Molybdenum; Pb: Lead; Sb: Antimony; TI: Thallium; HR: Hazard ratio; CI: Confidence intervals; BMI: Body mass index; PIR: Poverty income ratio.

Model 0: Adjusted for none.

Model 1: Adjusted for age, sex, race.

Model 2: Adjusted for age, sex, race, BMI, marital status, PIR, education, smoking, drinking, and health insurance.

Model 3: Adjusted for age, sex, race, BMI, marital status, PIR, education, smoking, drinking, health insurance, diabetes, stroke, cancer, CVD, and hypertension. *P*-value less than 0.025.

### The association between urinary metal and all-cause mortality by WQS regression model

**Fig 2:**
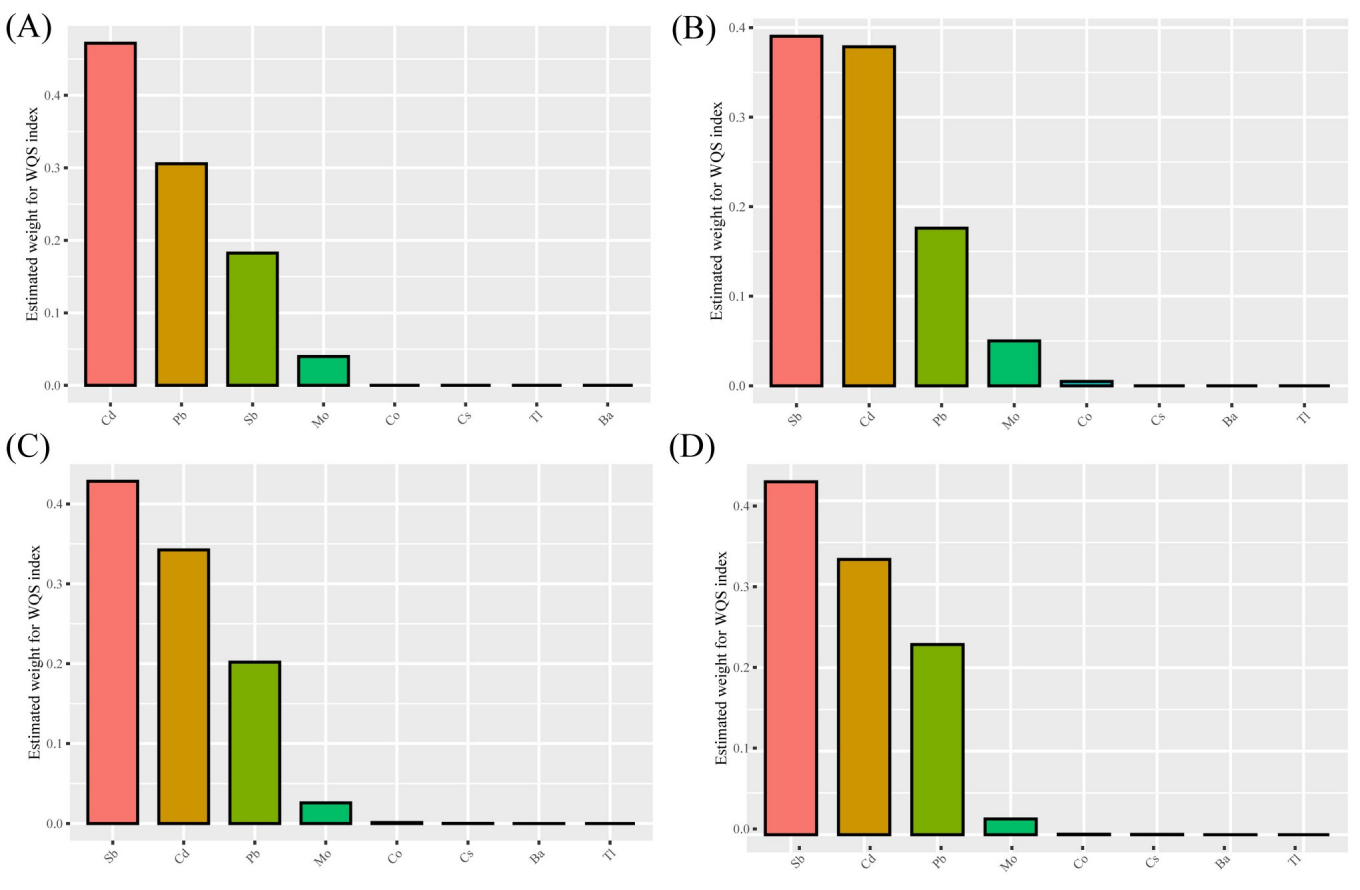
Weighted quantile sum (WQS) model regression index weights for all-cause mortality. **(**Ba: Barium; Cd: Cadmium; Co: Cobalt; Cs: Cesium; Mo: Molybdenum; Pb: Lead; Sb: Antimony; TI: Thallium; A: Adjusted for none; B: Adjusted for age, sex, race; C: Adjusted for age, sex, race, BMI, marital status, PIR, education, smoking, drinking, and health insurance; D: Adjusted for age, sex, race, BMI, marital status, PIR, education, smoking, drinking, health insurance, diabetes, stroke, cancer, CVD, and hypertension**)**.

The Model 3 of WQS analysis showed a positive association between metal mixtures (HR: 2.23; 95%CI: 2.00, 2.48) and all-cause mortality (Table 4). The weighted indices of the four metals (Sb, Cd, Pb, and Mo) in WQS are 0.356, 0.297, 0.262, and 0.080, respectively (Table 5). This result remained stable across Models 0, 1, and 2 (Fig 2).

**Table 4:** The WQS analysis of 8 metals and all-cause mortality.

	HR (95%CI)	*P*
**Model 0**		
mixed metals	3.20 (2.92,3.50)	< 0.001
**Model 1**		
mixed metals	2.48 (2.24,2.76)	< 0.001
**Model 2**		
mixed metals	2.23 (2.00,2.48)	< 0.001
**Model 3**		
mixed metals	2.23 (2.00,2.48)	< 0.001

HR: Hazard ratio; CI: Confidence intervals; BMI: Body mass index; PIR: Poverty income ratio; WQS: Weighted quantile sum.

Model 0: Adjusted for none.

Model 1: Adjusted for age, sex, race.

Model 2: Adjusted for age, sex, race, BMI, marital status, PIR, education, smoking, drinking, and health insurance.

Model 3: Adjusted for age, sex, race, BMI, marital status, PIR, education, smoking, drinking, health insurance, diabetes, stroke, cancer, CVD, and hypertension.

**Table 5:** Summary of all analysis results.

Metal	Cox regression	Competing risk model	WQS (positive direction)*	BKMR (PIP)^#^
Ba	/	-	<0.001	1.00
Cd	/	-	0.297	1.00
Co	+	+	<0.001	1.00
Cs	/	-	0.005	1.00
Mo	-	-	0.080	1.00
Pb	-	+	0.262	1.00
Sb	+	+	0.356	1.00
Tl	/	-	<0.001	1.00

Ba: Barium; Cd: Cadmium; Co: Cobalt; Cs: Cesium; Mo: Molybdenum; Pb: Lead; Sb: Antimony; TI: Thallium; WQS: Weighted quantile sum; BKMR: Bayesian kernel machine regression; PIP: Posterior inclusion probability.

+: Indicates that *P* < 0.05 in the final Cox model and Competing Risk model.

-: Indicates that *P* ≥ 0.05 in the final in the final Cox model and Competing Risk model.

/: Indicates that the Cox regression proportional hazards (PH) assumption is not met.

*: Indicates the weighted index in WQS, with values closer to 1 indicating a stronger association.

#: Indicates the PIP in the final BKMR model, with values closer to 1 indicating a stronger association.

### BKMR model to assess the association between urinary metal and all-cause mortality

**Fig 3:**
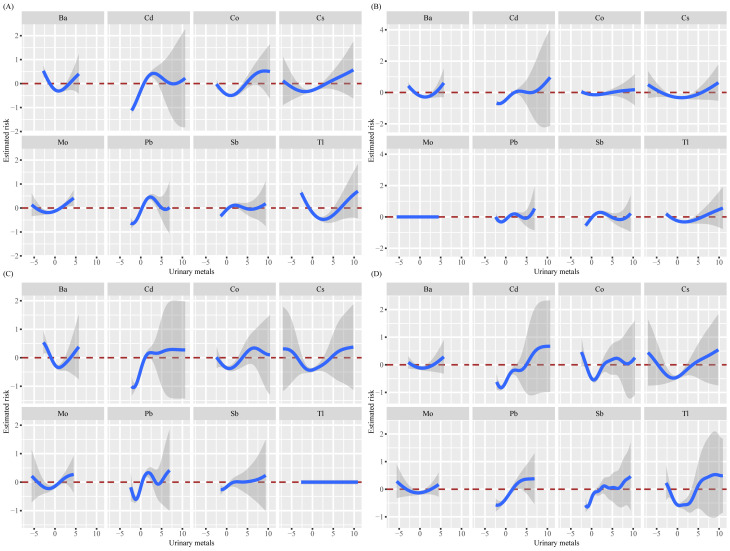
Univariate exposure-response function (95% CI) between urinary metals and all-cause mortality when fixing the concentrations of other metals at the median. (Ba: Barium; Cd: Cadmium; Co: Cobalt; Cs: Cesium; Mo: Molybdenum; Pb: Lead; Sb: Antimony; TI: Thallium; A: Adjusted for none; B: Adjusted for age, sex, race; C: Adjusted for age, sex, race, BMI, marital status, PIR, education, smoking, drinking, and health insurance; D: Adjusted for age, sex, race, BMI, marital status, PIR, education, smoking, drinking, health insurance, diabetes, stroke, cancer, CVD, and hypertension).

**Fig 4:**
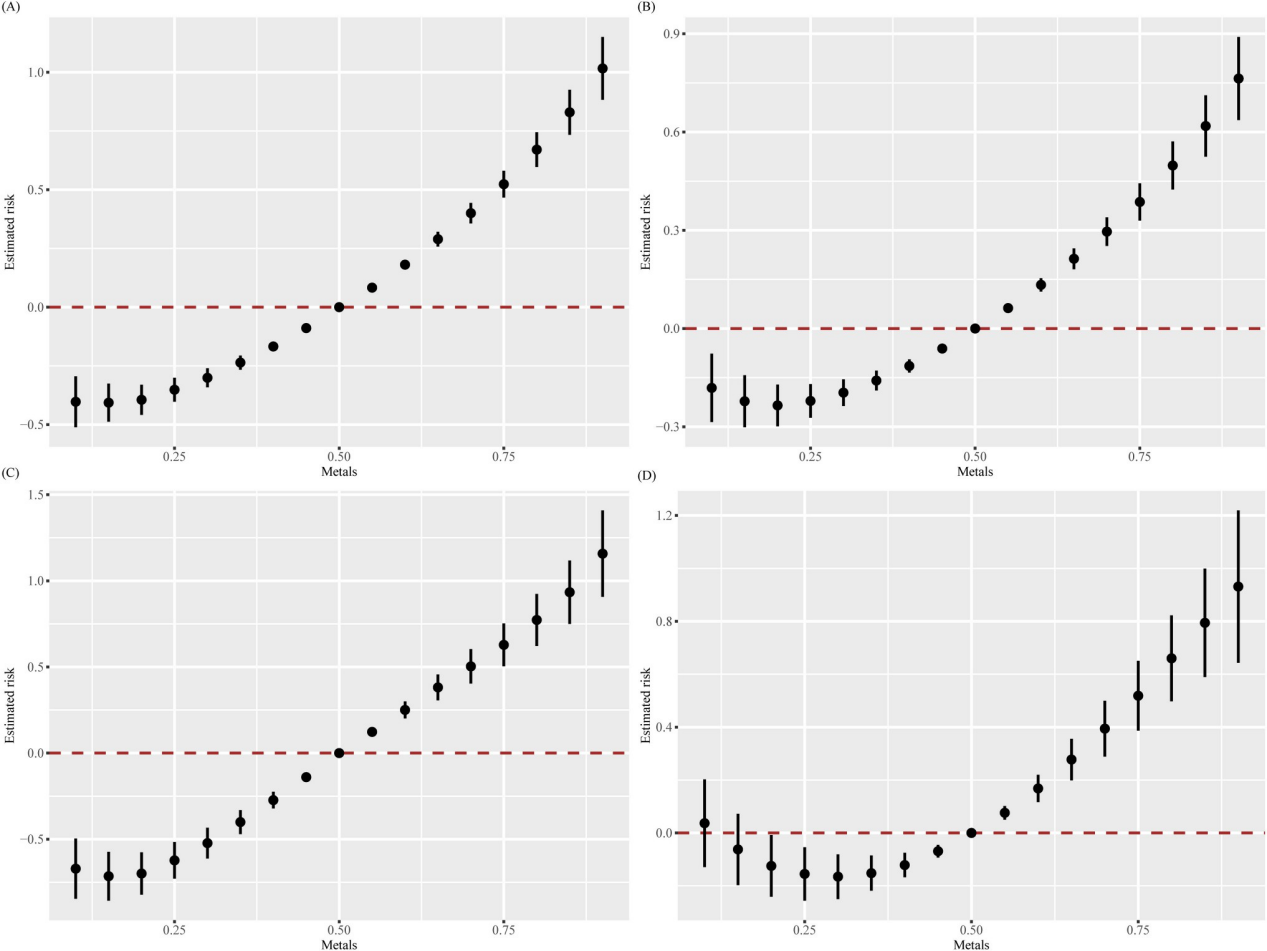
Joint effect of the mixture on all-cause mortality when all metals at particular percentiles were compared to all the metals at their 50th percentile by Bayesian kernel machine regression (BKMR) model. (A: Adjusted for none; B: Adjusted for age, sex, race; C: Adjusted for age, sex, race, BMI, marital status, PIR, education, smoking, drinking, and health insurance; D: Adjusted for age, sex, race, BMI, marital status, PIR, education, smoking, drinking, health insurance, diabetes, stroke, cancer, CVD, and hypertension).

In the final model, the relationship between all metals and all-cause mortality rate shows a complex non-linear relationship, with positive association within a certain range (Fig 3). Compared to the previous three models, it can be inferred that covariates have influenced the relationship between metal mixtures and all-cause mortality to some extent. We analyzed the overall impact of metal mixtures on all-cause mortality rate. Compared to the 50th percentile, when all metal mixtures are at or above the 40th percentile, there is an increasing trend in all-cause mortality rate (Fig 4). The posterior inclusion probabilities (PIPs) indicate that each metal is associated with all-cause mortality (Table 5).

## Discussion

In this research, we conducted a comprehensive comparison of the outcomes obtained from various statistical methods in order to assess the influence of 8 urinary metals on the mortality rate among the general population of the US. Firstly, Cox regression analysis showed a positive association between between Co and Sb with the all-cause mortality rate. Competing risk models provided additional evidence of a positive association between Co, Sb, Pb, and cardiovascular mortality. Sb, Pb, Cd, and Mo had the highest weight rankings in the final WQS model. All metals showed a complex non-linear relationship with all-cause mortality, with high PIPs in the final BKMR models.This study fully considered the strengths and limitations of different statistical methods and comprehensively evaluated the combined impact of urinary metals on mortality.

We observed that the detection rates of most metals increased over time, with all metals showing generally high detection rates except for Sb. Despite the lower detection rate of antimony, we found a significant association between it and mortality. This may suggest that antimony, even at low concentrations, can still have a notable impact on health, potentially due to its toxicity, bioaccumulation effects, or specific effects on certain populations. Therefore, the health risks of antimony should not be overlooked. We found an interaction Sb and certain years with respect to all-cause mortality (S5 Table). This suggests that the health impact of Sb may be modulated by temporal factors. Variations in Sb emissions, differences in detection technologies and methods, as well as socioeconomic factors and policy interventions in specific years may all influence this interaction.

Cox regression is most commonly used to assess the impact of chemical substances on all-cause and cardiovascular mortality. Typically, these analyses involve one or a group of similar chemical substances for ease of interpretation. However, metal exposures in real environments are not singular, and the interactions between mixed exposures may result in false positive or false negative results. After adjusting for all potential confounders, we found a positive association between Co, and Sb with overall mortality, which is consistent with previous research findings [32]. The epidemiological research on the relationship between adult cardiovascular diseases and environmental metals (such as Ba, Sb, etc.) is receiving increasing attention [33]. Some scholars have proposed that the traditional Cox regression overestimates the cumulative incidence rate when evaluating the impact of metals on cardiovascular mortality [34], so this analysis introduced a competitive risk model. We found that cobalt (Co), lead (Pb), and antimony (Sb) are significantly associated with an increased risk of cardiovascular mortality. The competing risk model has a distinct advantage over Cox regression in multiple potential events studies. It can accurately estimate the risk of a specific event while accounting for the impact of other competing events, thereby providing a more realistic risk assessment [16,27]. Previous literature has confirmed that the proportion of Cd and Sb in urine metal mixtures contributes the most to the incidence of cardiovascular diseases [35]. Sb is positively correlated with mean platelet volume (MPV), which contributes to thrombus formation and increases the risk of cardiovascular disease [1]. Additionally, literature has found that Co, Sb, and Pb have a significant impact on cardiovascular mortality rate [6]. The above results were consistent with our competition risk model results.

WQS analysis can better explain the complex impact of exposure on health in real life by considering the weight and correlation of chemical substances [30]. In our analysis, Sb, Cd, Pb, and Mo are considered to have the highest impact weight on overall mortality. On one hand, this may be due to differences in sample size, which results in a slight difference from the previous WQS regression analysis that mentioned cadmium having the highest weight in the mixture [6]. On the other hand, it has been demonstrated that Pb and Mo are significantly associated with all-cause mortality in the WQS regression model, possibly due to interactions with other metals. However, this association is not observed in the Cox regression model. This indicates that WQS analysis may be more sensitive in identifying important factors than individual analyses, but the standard WQS without penalty terms has limitations in simultaneously evaluating the joint effects of chemicals with different directions of action [36]. High Pb exposure has been confirmed to be positively correlated with the all-cause and cancer mortality [37,38]. Mo also has an independent and comprehensive correlation with all-cause mortality rate [7]. The results of this study once again provide additional evidence for the impact of mixed metal exposure on mortality.

The BKMR analysis can flexibly estimate multivariable exposure-response functions, identifying hidden complex patterns and non-linear relationships [31]. Our final model revealed a complex non-linear relationship between 8 types of metal substances and overall mortality rate, with a positive correlation within a certain range. Due to the introduction of kernel functions, the calculation complexity of the BKMR model is high, leading to slower computation speed and higher requirements for computer configuration. It is necessary to choose appropriate kernel functions and related parameters based on actual situations in order to ensure better interpretability of the model.

This study demonstrates that traditional survival analysis can provide a simple relationship between individual metals and mortality. When considering competing biases, the competing risk model is an alternative additional strategy [39]. The WQS model can explore the impact of mixed exposures on the target outcome [40]. The BKMR model can explore the exposure-response effects of each substance and the interactions between substances [41]. Therefore, these models have their own advantages and disadvantages, and their combined application is beneficial for a comprehensive assessment of the effects of mixed substances on the outcome. The main findings of this study are summarized in Table 5, which can provide a reference for future related research.

Our analysis has some limitations. The assumption underlying our analysis is that the sample based on inclusion analysis represents the adult population of the US. Nevertheless, it remains unclear whether the WQS and BKMR models can be applied under this assumption. In addition, we referred to previous designs that used unweighted data as unweighted estimates are preferable to weighted estimates if the covariates used to calculate sample weights are already included in the regression model [42]. Another limitation is the use of cross-sectional survey data. The measured metal levels in this study can only reflect recent exposure, and long-term follow-up is needed to determine the actual impact of metals on mortality outcomes. Additionally, we did not discuss the relationship between mixed metals and cancer mortality. Furthermore, we cannot establish a causal relationship between mixed metals and mortality. To gather more evidence, it will be necessary to conduct large-scale prospective cohort studies or experimental studies in the future.

## Conclusion

Combining all models, it is possible that Sb may have a more stable impact on overall mortality and cardiovascular mortality. We recommend using multiple methods to comprehensively explain the effects of mixtures, meaningful metal effects in individual statistical models still require careful attention.

## Supporting information

S1 Fig Cumulative incidence of all-cause and cardiovascular death events. (DOCX)

S2 Fig Pairwise Pearson correlations between 8 metal concentrations. (DOCX)

S3 Fig Concentration trends of 8 metals (ln-transformed) during each survey period. (DOCX)

S1 Table Proportional hazard assumption in the Cox model. (DOCX)

S2 Table Distributions of metals in the study population. (DOCX)

S3 Table The relationship between 8 metals and survey period. (DOCX)

S4 Table The goodness-of-fit metrics for the final Cox model. (DOCX)

S5 Table Pb*cycle interaction in the crude Cox regressions. (DOCX)

S1 Data This file contains the raw data used for statistical analysis in the manuscript. (CSV)
