# Supplementary material for: Association between exposure to urinary metal and all-cause and cardiovascular mortality in US adults
Source: PLoS One. 2024 Dec 27;19(12):e0316045. doi: 10.1371/journal.pone.0316045 (PMC11676533; doi:10.1371/journal.pone.0316045)
Supplement: S1 Fig — (DOCX) [file pone.0316045.s001.docx]

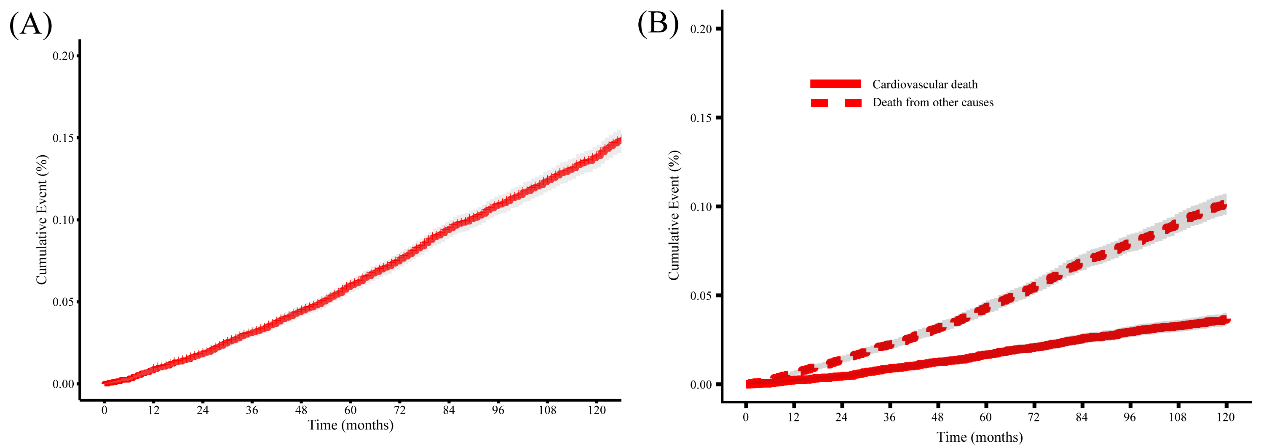


Fig S1. Cumulative incidence of all cause and cardiovascular death events.（A: all-cause mortality; B: cardiovascular mortality）
