# Supplementary material for: Association between exposure to urinary metal and all-cause and cardiovascular mortality in US adults
Source: PLoS One. 2024 Dec 27;19(12):e0316045. doi: 10.1371/journal.pone.0316045 (PMC11676533; doi:10.1371/journal.pone.0316045)
Supplement: S3 Fig — (DOCX) [file pone.0316045.s003.docx]

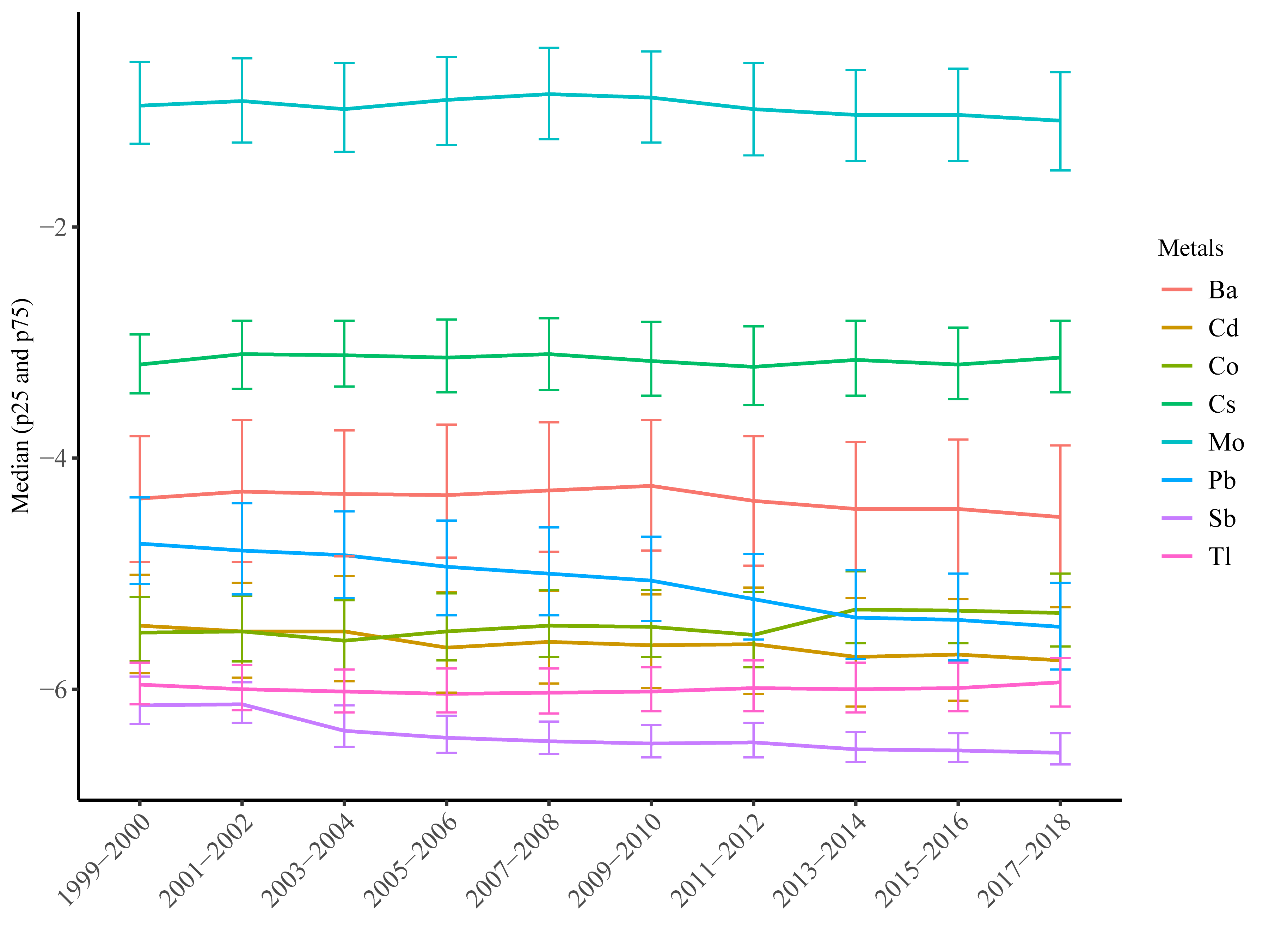


Fig S3. Concentration trends of 8 metals(ln-transformed) during each survey period (median (P25 and P75), Ba: barium; Cd: cadmium; Co: cobalt; Cs: cesium; Mo: molybdenum; Pb: lead; Sb: antimony; TI: thallium.).
