# Supplementary material for: Association between exposure to urinary metal and all-cause and cardiovascular mortality in US adults
Source: PLoS One. 2024 Dec 27;19(12):e0316045. doi: 10.1371/journal.pone.0316045 (PMC11676533; doi:10.1371/journal.pone.0316045)
Supplement: S1 Table — (DOCX) [file pone.0316045.s004.docx]

Table S1. Proportional hazard assumption in Cox model

| Metals | Chisq | *P* |
| --- | --- | --- |
| Ba | 14.90 | 0.00 |
| Cd | 8.34 | 0.00 |
| Co | 1.09 | 0.30 |
| Cs | 4.40 | 0.04 |
| Mo | 1.32 | 0.25 |
| Pb | 0.95 | 0.33 |
| Sb | 0.13 | 0.72 |
| Tl | 7.51 | 0.01 |
| Ba: barium; Cd: cadmium; Co: cobalt; Cs: cesium; Mo: molybdenum; Pb: lead; Sb: antimony; TI: thallium. | | |
