# Supplementary material for: Association between exposure to urinary metal and all-cause and cardiovascular mortality in US adults
Source: PLoS One. 2024 Dec 27;19(12):e0316045. doi: 10.1371/journal.pone.0316045 (PMC11676533; doi:10.1371/journal.pone.0316045)
Supplement: S2 Table — (DOCX) [file pone.0316045.s005.docx]

Table S2. Distributions of metals in the study population

| **Metals (μg/L)** | **Detection rate (%)** | **Median** | **Interquartile range** |
| --- | --- | --- | --- |
| Ba | 98.81 | 1.19 | 0.59, 2.31 |
| Cd | 94.87 | 0.26 | 0.13, 0.52 |
| Co | 99.69 | 0.36 | 0.22, 0.57 |
| Cs | 99.99 | 4.55 | 2.74, 6.82 |
| Mo | 99.98 | 41.00 | 21.40, 70.00 |
| Pb | 97.36 | 0.50 | 0.27, 0.92 |
| Sb | 72.99 | 0.06 | 0.03, 0.11 |
| Tl | 99.48 | 0.16 | 0.09, 0.25 |
| Ba: barium; Cd: cadmium; Co: cobalt; Cs: cesium; Mo: molybdenum; Pb: lead; Sb: antimony; TI: thallium. | | | |
