# Supplementary material for: Association between exposure to urinary metal and all-cause and cardiovascular mortality in US adults
Source: PLoS One. 2024 Dec 27;19(12):e0316045. doi: 10.1371/journal.pone.0316045 (PMC11676533; doi:10.1371/journal.pone.0316045)
Supplement: S3 Table — (DOCX) [file pone.0316045.s006.docx]

Table S3. The relationship between 8 metals and survey period

| Metals | *P* for trend |
| --- | --- |
| Ba | 0.802 |
| Cd | <0.001 |
| Co | 0.134 |
| Cs | 0.021 |
| Mo | 0.005 |
| Pb | <0.001 |
| Sb | <0.001 |
| Tl | <0.001 |
| Ba: barium; Cd: cadmium; Co: cobalt; Cs: cesium; Mo: molybdenum; Pb: lead; Sb: antimony; TI: thallium. | |
