# Supplementary material for: Association between exposure to urinary metal and all-cause and cardiovascular mortality in US adults
Source: PLoS One. 2024 Dec 27;19(12):e0316045. doi: 10.1371/journal.pone.0316045 (PMC11676533; doi:10.1371/journal.pone.0316045)
Supplement: S4 Table — (DOCX) [file pone.0316045.s007.docx]

Table S4. The goodness-of-fit metrics for final cox model

| Metals | Adjusted model (R^2^) |
| --- | --- |
| Co | 0.808 |
| Mo | 0.806 |
| Pb | 0.807 |
| Sb | 0.807 |
| Co: cobalt; Mo: molybdenum; Pb: lead; Sb: antimony. | |
