# Supplementary material for: Association between exposure to urinary metal and all-cause and cardiovascular mortality in US adults
Source: PLoS One. 2024 Dec 27;19(12):e0316045. doi: 10.1371/journal.pone.0316045 (PMC11676533; doi:10.1371/journal.pone.0316045)
Supplement: S5 Table — (DOCX) [file pone.0316045.s008.docx]

Table S5. Pb*cycle interaction in the crude Cox regressions

| Variable | HR | 95% CI | *P* |
| --- | --- | --- | --- |
| Pb*Year2001-2002 | 1.21 | 1.21(0.95, 1.55) | 0.12 |
| Pb*Year2003-2004 | 1.44 | 1.44(1.12, 1.86) | 0.01 |
| Pb*Year2005-2006 | 1.34 | 1.34(1.05, 1.71) | 0.02 |
| Pb*Year2007-2008 | 1.23 | 1.23(0.96, 1.58) | 0.10 |
| Pb*Year2009-2010 | 1.31 | 1.31(1.02, 1.70) | 0.04 |
| Pb*Year2011-2012 | 1.08 | 1.08(0.83, 1.41) | 0.57 |
| Pb*Year2013-2014 | 0.88 | 0.88(0.64, 1.21) | 0.43 |
| Pb*Year2015-2016 | 1.12 | 1.12(0.77, 1.64) | 0.54 |
| Pb*Year2017-2018 | 2.08 | 2.08(1.07, 4.04) | 0.03 |
| Pb: lead; HR: hazard ratio; CI: confidence intervals. | | | |
